# Zinc Therapy in Dermatology: A Review

**DOI:** 10.1155/2014/709152

**Published:** 2014-07-10

**Authors:** Mrinal Gupta, Vikram K. Mahajan, Karaninder S. Mehta, Pushpinder S. Chauhan

**Affiliations:** Department of Dermatology, Venereology & Leprosy, Dr. R. P. Govt. Medical College, Kangra (Tanda), Himachal Pradesh 176001, India

## Abstract

Zinc, both in elemental or in its salt forms, has been used as a therapeutic modality for centuries. Topical preparations like zinc oxide, calamine, or zinc pyrithione have been in use as photoprotecting, soothing agents or as active ingredient of antidandruff shampoos. Its use has expanded manifold over the years for a number of dermatological conditions including infections (leishmaniasis, warts), inflammatory dermatoses (acne vulgaris, rosacea), pigmentary disorders (melasma), and neoplasias (basal cell carcinoma). Although the role of oral zinc is well-established in human zinc deficiency syndromes including acrodermatitis enteropathica, it is only in recent years that importance of zinc as a micronutrient essential for infant growth and development has been recognized. The paper reviews various dermatological uses of zinc.

## 1. **Introduction**

Zinc, a divalent cation, is an essential micronutrient for humans and its importance can be gauged from the fact that it is an essential component of more than 300 metalloenzymes and over 2000 transcription factors that are needed for regulation of lipid, protein and nucleic acid metabolism, and gene transcription. It is involved in gene transcription at various levels, via participation in histone deacetylation reactions and via factors possessing the zinc-finger motifs [1]. An important family of zinc-finger proteins is the steroid or thyroid hormone receptors that bind hormones and facilitate their wide range of effects. Zinc also plays an important role in maintaining the proper reproductive function, immune status, and wound repair via regulation of DNA and RNA polymerases, thymidine kinase, and ribonuclease. It maintains macrophage and neutrophil functions, natural killer cell activity, and complement activity. It activates natural killer cells and phagocytic function of granulocytes and stabilizes the plasma subcellular membranes especially the lysosomes. It inhibits the expression of integrins by keratinocytes and modulates the production of TNF-*α* and IL-6 and reduces the production of inflammatory mediators like nitric oxide. It is also proposed that it is toll-like receptors mediated regulation of zinc homeostasis which influences dendritic cell function and immune processes [2]. Zinc also possesses antioxidant property and has been found useful in preventing UV-induced damage and reducing the incidence of malignancies. It has also been demonstrated to possess antiandrogenic properties as it causes modulation of 5*α*-reductase type 1 and 2 activity [1, 3, 4].

## 2. **Zinc Physiology and Zinc Deficiency States**

It will be prudent to revisit the physiological aspects of zinc metabolism before discussing zinc deficiency states. Briefly, an average adult weighing 70 kg has a body zinc content of 1.4–2.3 gm, the highest tissue concentration (>500 *μ*g/g dry weight) being in the prostate, seminal fluid, uveal tissue, and skin. While about half of the total body zinc is in the bones, the skin contains nearly 6% of total body zinc. As movement of zinc across various tissues is limited and there is no storage depot, the continuous external supply of zinc is important for metabolic needs, growth, and tissue repair. The recommended daily allowance of zinc for an average adult male is 11 mg and the requirement increases from 8 mg/d to up to 12 mg/d in females during pregnancy and lactation. Animal foods like meat, eggs, fish, and oysters are rich in zinc. Although cereals and legumes contain moderate amount of zinc, only 20–40% of the ingested metal is absorbed. Its absorption is hampered by the presence of phytates, calcium, and phosphates while chelating agents like EDTA and animal proteins increase its absorption from gut. Zinc is mainly absorbed from proximal jejunum and distal duodenum and is perhaps facilitated by the presence of low molecular weight zinc binding ligands. It is excreted mainly through feces and in small amounts in urine and sweat.

Zinc deficiency is a common problem with an estimated 1/3rd of world population suffering from zinc deficiency and is highly prevalent in Southeast Asia, sub-Saharan Africa, and other developing countries [5]. Zinc deficiency can be from inadequate dietary intake and poor absorption or because of increased loss. Endemic zinc deficiency occurring in rural Iran, Egypt, and Turkey has been attributed to eating whole grain bread with high fibre and phytate contents that render zinc nearly unabsorbable. Poor-socioeconomic status, protein calorie malnutrition, protein restricted and vegetarian diets, anorexia nervosa, exclusive parenteral nutrition, chronic gastrointestinal diseases, hookworm infestation and malabsorption syndromes, pancreatic insufficiency, chronic renal failure or malignancies, infants on formula milk with low zinc or parenteral alimentation, and acrodermatitis enteropathica are some of the predisposing factors for poor availability and/or absorption of zinc.

## 3. **Hypozincemia of Infancy**

Zinc is now well-recognized micronutrient essential for infant growth and development and is a standard component in parenteral nutrition for infants with low birth weight or chronic gastrointestinal dysfunction. Some researchers have differentiated hypozincemia of infancy in three categories: type-1 or classic acrodermatitis enteropathica is a rare genetic disorder of zinc deficiency because of mutations in zinc transporter genes, type-2 or due to defective secretion of zinc in mother's milk, and type-3 or hypozincemia in preterm infants on prolonged parenteral alimentation. Type-1 or classic acrodermatitis enteropathica is autosomal recessive disorder while type-2 hypozincemia is perhaps inherited as an autosomal recessive or x-linked disorder. Type-3 or hypozincemia in preterm infants is temporary and occurs from deficient low body reserves due to prematurity or parenteral nutrition deficient in zinc. The clinical manifestations are mainly due to low zinc levels and are similar in all three types and improvement is usually rapid on initiation of zinc therapy.

## 4. **Acrodermatitis Enteropathica**

Acrodermatitis enteropathica is a rare disease with an estimated prevalence of 1 in 500000 people in Denmark. The exact cause of poor zinc absorption is poorly understood but picnolic acid, a tryptophan derivative, has been implicated as the deficient ligand. The onset of symptoms is usually seen around 4–6 weeks after weaning or even earlier in infant not on breast milk. The infant becomes irritable and withdrawn and develops photophobia. Anorexia, pica, growth impairment, hypogonadism, impaired taste and smell, night blindness, and neuropsychiatric symptoms (mood changes, tremors, dysarthria, and jitteriness) eventuate in untreated cases. Cutaneous changes include periorificial and acral dermatitis (some lesions are burn like, oozy, or psoriasiform) localized around mouth, cheeks, ears, nostrils, buttocks, anus, dorsal skin of hands, feet, fingers, toes, and heels, paronychia, nail dystrophy, and hair loss. Delayed wound healing, angular stomatitis, conjunctivitis, blepharitis, increased susceptibility to infection, and growth retardation may also be seen. Low serum alkaline phosphatase and zinc levels (<50 *μ*g%) are diagnostic and will improve after zinc therapy but hypoalbuminemia does not necessarily indicate zinc deficiency. Treatment with oral zinc (2-3 mg/kg/day) will cure all clinical manifestations within 1-2 weeks and needs to be continued up to adulthood for continuous supplementation and favorable long-term prognosis.

## 5. **Zinc Therapy in Dermatology**

Zinc, elemental or in its various forms (salts), has been used as a therapeutic modality for centuries. Topical preparations like zinc oxide, calamine, or zinc pyrithione have been in use as photoprotecting, soothing agents or as active ingredient of antidandruff shampoos. Its use has also expanded manifold over the years for a number of dermatological conditions including infections (warts, leishmaniasis), inflammatory dermatoses (acne vulgaris, rosacea), pigmentary disorders (melasma), and neoplasias (basal cell carcinoma). Although the role of oral zinc is well-established in human zinc deficiency syndromes including acrodermatitis enteropathica, it is only in recent years that importance of zinc as a micronutrient essential for infant growth and development has been recognized. We review here various therapeutic uses of both topical and oral zinc in dermatology clinical practice (Table 1).

## 6. **Infections**

Zinc, alone or as an adjuvant, has been found useful in many dermatological infections owing to its modulating actions on macrophage and neutrophil functions, natural killer cell/phagocytic activity, and various inflammatory cytokines.

### 6.1. Warts

Warts are a common dermatological human papilloma virus infection with numerous available treatment modalities. Over the years, various destructive procedures performed accurately have remained the only effective remedy for common warts. Zinc can be a useful topical or oral treatment modality in common warts as many studies have demonstrated efficacy of both oral and topical zinc in treating warts without significant adverse effects. Sharquie et al. [6] studied the efficacy of topical zinc sulphate in viral warts. In their pilot clinical trial 10 patients with plane warts were treated with 10% zinc sulphate solution applied thrice daily for a period of 4 weeks. They observed complete clearance in 80% patients. The authors also reported results of double blind clinical trial in the same study comprising 50 patients with common warts and 40 patients with plane warts treated with topical 10% and 5% zinc sulphate solution applied thrice daily for 4 weeks while distilled water was used as a control [6]. Complete clearance of plane warts was seen in 85.7% and 42.8% cases from topical 10% and 5% zinc sulphate solution, respectively, while complete clearance was also seen in 11% and 5% of patients with common warts using 10% and 5% zinc sulphate solution, respectively, which was statistically insignificant. Khattar et al. [7] observed that topical 20% zinc oxide was more effective than ointment containing salicylic acid (15%) and lactic acid (15%) for the treatment of warts in a randomized double-blind controlled trial of 44 patients. A complete cure was observed after 3 months in 50% patients with common warts in zinc oxide group as compared to 42% in the other group. Oral zinc sulphate (10 mg/kg/day) given for 2 months in common warts also resulted in complete clearance in 61% patients in one month and 87% after two months of therapy in a placebo controlled trial by Al-Gurairi et al. [8], whereas clearance rate was 50% with the same dose of oral zinc sulphate after 2 months in an open-label clinical study by Mun et al. [9]. Oral zinc (10 mg/kg/day) has been reported to clear recalcitrant warts in a patient with epidermodysplasia verruciformis in 12 weeks [10]. Intralesional 2% zinc sulphate too has been found to induce clearance of warts [11]. Topical or oral zinc can be a useful therapeutic modality for warts especially in children wherein painful physical treatment options have limited usefulness.

### 6.2. Cutaneous Leishmaniasis

Cutaneous leishmaniasis is a major health problem that causes significant morbidity due to long clinical course and residual scarring. It is caused by parasites of* Leishmania* spp. It occurs worldwide especially in countries having tropical climatic conditions and is significant problem among returning travelers. It has been estimated that 1.5 million new cases of cutaneous leishmaniasis occur annually with majority of them being reported in Brazil, Iran, and Afghanistan. New endemic foci too are being recognized world over in recent years. Although pentavalent antimonials remain the drug of choice despite concerns for cardiac and renal toxicity, search for safer and effective alternative drugs continues. Many other agents including zinc have been tried with variable success. Zinc, both intralesionally and orally, has been found effective in the management of cutaneous leishmaniasis. Iraji et al. [12], in a prospective, double blind, case-control clinical study, observed a comparable response rate with intralesional 2% zinc sulphate and meglumine antimoniate after six weeks of therapy. Sharquie et al. [13] used oral zinc sulphate in doses of 2.5, 5, and 10 mg/kg/day for 45 days among 104 patients with cutaneous leishmaniasis and observed cure rates of 83.9%, 93.1%, and 96.9% for the 2.5 mg/kg, 5 mg/kg, and 10 mg/kg treatment groups, respectively, without significant adverse effects. Although low cost and better safety profile of oral zinc sulphate as compared to antimonials appears attractively advantageous, inconsistent outcome remains a limiting factor for its solo use.

### 6.3. Leprosy

Leprosy is a chronic infection of skin and peripheral nerves caused by* Mycobacterium leprae*. Almost 4 million people have been affected by leprosy and nearly 250,000 new cases are still being detected annually throughout the world. WHO recommended multidrug therapy (MDT) which has been the mainstay of treatment for leprosy and in reducing its prevalence to near elimination levels. However, lepra reactions and nerve damage cause significant morbidity among patients affected with leprosy. Apart from well-established treatment with systemic corticosteroids and thalidomide, and many anti-inflammatory and immunomodulator drugs, oral zinc has been found useful in the management of lepra reactions owing to its immunostimulatory properties. Zinc is found to stimulate production of IL-2 and induces a shift from Th2 to Th1 response. It has also been demonstrated to decrease the serum levels of TNF-*α* and inhibit the TNF-*α* induced apoptosis of peripheral blood mononuclear cells that helps in controlling the disease activity and reactional states [14]. In a study comprising patients of recurrent erythema nodosum leprosum additionally receiving zinc, the steroids could be tapered off completely and the duration and severity of reaction were also reduced [15]. Addition of oral zinc to antileprosy treatment too has been shown to improve therapeutic outcome. Oral zinc when given as an adjuvant to dapsone in lepromatous leprosy induced rapid lepromin conversion and bacterial clearance in the patients as compared to the control group. The clinical improvement was also faster in patients receiving zinc as an adjuvant along with standard MDT [16]. Oral zinc perhaps makes an adjuvant of choice in leprosy treatment.

### 6.4. Herpes Genitalis

Herpes genitalis, caused by Herpes simplex virus (HSV) 1 and 2, is characterized by a high rate of recurrences.* In vivo* use of zinc acetate gel has been found effective in preventing sexual transmission of HSV-2 and HIV infections [17]. Mahajan et al. [18] used zinc sulphate as 1%, 2%, and 4% topically in three groups of 30 patients each with herpes genitalis for a period of 3 months and observed that higher concentrations were more effective in treating as well as preventing recurrences. However, not many studies are available for making any recommendations.

### 6.5. Dermatophytoses

Dermatophytoses are a diverse array of disorders involving the skin, hair, and nails. Antifungals agents like azoles and allylamines, both topical and systemic, form the mainstay of treatment. Of late, emergence of resistance to these agents is being observed in clinical practice when a need for different/additional treatment modalities is being felt. Zinc in combination with 2% undecylenic acid has been tried for the treatment of dermatophytoses. Chretien et al. [19] in a randomized control trial in 151 patients of tinea pedis studied the efficacy of a powder containing 20% zinc-undecylenate, 2% undecylenic acid, and a placebo powder. After a study period of 4 weeks, a negative culture and a negative KOH examination were seen in 88% and 80% as compared to 17% and 49% in placebo group. Clinical improvement in the form of decreased erythema, scaling, and itching was also significantly more in the former group.

### 6.6. Bromhidrosis

Bromhidrosis is a common disorder characterized by foul smelling sweat. A strong odor is usually associated with increased bacterial flora, usually of* Corynebacterium* sp. Topical antibacterials and antiperspirants are the treatment of choice along with maintenance of good hygiene. Owing to its antibacterial action, topical zinc sulphate has been tried and found effective in the management of axillary bromhidrosis and plantar malodor [20, 21]. Sharquie et al. [21] in a single blinded placebo controlled therapeutic trial studied the efficacy of 15% zinc sulphate solution for foot malodor. Zinc sulfate solution 15% was applied to sole and toe-webs once daily for two weeks and three times per week for next two weeks followed by single application weekly as maintenance after clearance of odor for two months. Fifty-eight patients received zinc sulphate solution while other 50 patients received a placebo solution. Thirty-five of the 50 (70%) patients who completed the study showed complete clearance of foot odor as compared to only 1 (2%) subject in placebo group and the difference was statistically significant.

### 6.7. Pityriasis Versicolor

Pityriasis versicolor is a common fungal disorder presenting as truncal hypopigmented scaly macules. It is a common condition in tropics and may affect up to 40% of the population. Azole antifungals, like itraconazole and ketoconazole, both in topical and systemic formulations, form the mainstay of treatment. Zinc pyrithione 1% is a proven treatment modality for pityriasis versicolor owing to its anti-inflammatory action and direct cytotoxic action on* Pityrosporum ovale*. Topical zinc sulphate too has been used for the management of pityriasis versicolor. Sharquie et al. [22] observed complete clinical and mycological cure after 3 weeks' treatment with once daily application of 15% topical zinc sulphate in their single blinded placebo controlled study comprising 30 patients with pityriasis versicolor while no patient in placebo group showed any response.

## 7. **Inflammatory Dermatoses**

The anti-inflammatory properties of zinc have been the reasons for its use in many common inflammatory dermatoses like acne, rosacea, eczemas, and ulcers and wounds of varied etiology.

### 7.1. Acne Vulgaris

Acne vulgaris is the most common disorder among the adolescent age group affecting 90–95% of the midteen population. A large variety of topical and systemic agents is used for their management. Oral and topical antibiotics and/or retinoids are the commonly used therapies. A chronic persistent clinical course along with the emergence of resistance to common antibiotics has led to trial of numerous novel agents in acne management. Zinc has been used extensively both topically and systemically for the management of acne vulgaris since the time its favorable effect on acne was recognized by Michaelsson in a patient of acrodermatitis enteropathica and subsequent studies demonstrated low serum zinc in acne patients [23–28]. Although topical zinc sulfate was not effective and caused significant local irritation, the efficacy of topical antiacne medications containing zinc acetate or octoate with or without erythromycin is either equal or superior to erythromycin, tetracycline, or clindamycin used alone in reducing the severity of acne and the number of lesions [29–33]. Contrarily, Sharquie et al. [34] observed beneficial effect of topical 5% zinc sulfate in a single-blinded randomized study in 47 patients with mild acne vulgaris comparing it with topical 2% tea lotion. Although zinc appears to enhance the topical absorption of erythromycin in a study, the onset of action of erythromycin with zinc acetate applied twice daily was slower than benzoyl peroxide with clindamycin phosphate used once daily while overall efficacy and adverse effects were similar in another study [35, 36]. Oral zinc sulfate is reportedly more effective in the treatment of severe acne than for the treatment of mild to moderate acne but nausea, vomiting, and diarrhea occur frequently [37–40]. Similarly, oral zinc gluconate has been found useful in managing inflammatory acne but the initial loading dose is not beneficial [41–43]. However, acne treatment with zinc salts appears to be equal or less effective compared with systemic tetracyclines (minocycline, oxytetracycline) [40, 42, 44]. Recently a methionine bound zinc complex with antioxidants has been tried and found useful in managing mild to moderate acne vulgaris [45]. Zinc, with or without nicotinamide, is also another emerging alternate acne treatment to reduce possible adverse effects of antibiotics and in view of* Propionibacterium acnes* strains developing resistance to conventional antibiotics [46]. The exact mechanism of zinc in acne treatment remains poorly elucidated and is considered to act directly on microbial inflammatory equilibrium and facilitate antibiotic absorption when used in combination. Topical zinc alone as well as in combination with other agents is effective perhaps because of its anti-inflammatory activity and ability to reduce* P*.* acnes* counts by inhibition of* P*.* acnes* lipases and free fatty acid levels [37]. Another proposed mechanism for the benefit of zinc in acne is suppression of sebum production by its antiandrogenic activity [47].

### 7.2. Rosacea, Hidradenitis Suppurativa, Acne Conglobata, and Folliculitis Decalvans

Rosacea is a chronic disorder characterized by frequent flushing, erythema, and telangiectasia, interspersed by episodes of inflammation during which swelling, papules, and pustules are seen. A number of drugs, which include antibiotics (tetracyclines, metronidazole), immunosuppressants (calcineurin inhibitors-tacrolimus, pimecrolimus), retinoids, and vascular lasers, are the commonly used treatments. Oral zinc sulphate was found useful in the management of rosacea by Sharquie et al. [48]. They used zinc sulphate 100 mg thrice daily in 25 patients of rosacea in a double blind randomized control trial and observed a statistically significant decrease in disease activity after three months of therapy without any serious adverse effects. However, Bamford et al. [49] observed no significant improvement with oral zinc therapy in another randomized control trial. The antioxidant and anti-inflammatory properties of zinc have been postulated to be useful in the management of rosacea. The anti-inflammatory and antioxidant actions of zinc have also been utilized for the management of other follicular occlusion disorders like hidradenitis suppurativa, acne conglobata, and folliculitis decalvans as well. Brocard et al. [50] observed clinical response without significant side effects in all 22 patients of hidradenitis suppurativa when treated with zinc gluconate 90 mg/day. Similarly, Kobayashi et al. [51] reported complete cure of acne conglobata and dissecting cellulitis with oral zinc sulphate. However, overall benefit of zinc in these disorders remains understudied.

### 7.3. Psoriasis and Psoriatic Arthritis

Psoriasis is a common disorder affecting nearly 2-3% of general population with joint involvement being a common disabling complication. A large armamentarium of drugs ranging from time tested modalities like coal tar and phototherapy, methotrexate, and retinoids to newer “biological” modalities are being currently used. However, the chronically relapsing nature of the disease has always compelled the researchers to look for novel and safe therapies. Zinc has been tried for the management of psoriasis and psoriatic arthritis. Sadeghian et al. [52] found topical 0.25% zinc pyrithione cream, applied twice daily, effective for localized plaque psoriasis in a randomized double-blind controlled trial. The benefit was attributed to antiproliferative effect of zinc pyrithione. Oral zinc sulphate was found effective for psoriatic arthritis by Clemmensen et al. [53] in a double blind crossover trial versus placebo in 24 patients of psoriatic arthritis. However, oral zinc sulphate did not produce clinically significant improvement as a treatment modality for plaque psoriasis [54].

### 7.4. Eczemas

Eczemas comprise a diverse group of dermatoses with variable etiology and clinical manifestations and constitute significant proportion of all dermatological diseases with an estimated prevalence of 18 cases per 1000 US population. Contact dermatitis of occupational origin is by far the most common form of eczema and hand eczema accounts for majority of the cases. Depending upon the principal causative factors, the eczema may be endogenous eczema (atopic dermatitis, seborrhoeic dermatitis, discoid or nummular eczema, and asteatotic eczema) or exogenous or contact eczema (allergic or irritant contact dermatitis, photoallergic contact dermatitis). However, the clinical presentation of eczema may be modified by regional variation in skin structure and function such as in case of hand eczema. Apart from removal of the etiological agent, use of immunosuppressants like corticosteroids and calcineurin inhibitors form the mainstay of treatment. Zinc has anti-inflammatory properties and increases reepithelialization supporting its use for treating eczemas. Zinc oxide paste has been used for the treatment of diaper dermatitis since long. Although it is less effective as compared to other treatment modalities like topical corticosteroids, it is a useful soothing and antipruritic agent [55, 56]. A statistically significant improvement was observed with a combination cream containing zinc sulphate (2.5%) and clobetasol (0.05%) over plain clobetasol (0.05%) cream in 47 patients of chronic hand eczema in a double-blind, right to left, prospective clinical trial by Faghihi et al. [57]. Topical zinc oxide for its strong antioxidant and antibacterial action has been also used in treating atopic dermatitis, a chronic inflammatory eczematous dermatosis characterized by the impairment of the skin-barrier function, increased oxidative cellular stress, and bacterial colonization. Zinc oxide impregnated textiles have been tried* in vivo* for the management of atopic dermatitis in a study and a significant improvement was observed in the disease severity, pruritus, and subjective sleep in patients who wore zinc oxide-impregnated textiles than in control group [58]. These zinc oxide-functionalized textiles could be the upcoming treatment modality of choice for atopic dermatitis for future.

### 7.5. Ulcers and Wounds

Ulcers of variable etiology are a common presentation in the dermatology outpatients with an estimated community prevalence of 0.2%. Ulcer management is a challenging task for the treating physician as poor response to treatment is frequent in a sizeable proportion of cases due to the persistence of underlying etiological factors. Zinc, both oral and topical, for its healing properties has been used for a long time for the management of ulcers and wounds of varied etiology. Although oral zinc sulphate was initially reported to enhance the healing of arterial/venous leg ulcers, recent systematic reviews and meta-analysis have found no statistically significant response [59]. However, topical preparations containing zinc oxide have been used in the management of arterial and venous leg ulcers, pressure ulcers, and diabetic foot ulcers. The reported response rate was 83% in a study on efficacy of topical zinc oxide paste in both arterial and venous ulcers [60]. Usefulness of zinc iontophoresis has been demonstrated in ischemic skin ulcers as well [61]. Sehgal et al. [62] used phenytoin sodium, zinc oxide paste in 40 leprosy patients with trophic ulcers. After 4 weeks of daily therapy, 55% patients showed complete clearance of the ulcers while 82.5% showed development of granulation tissue. This therapeutically beneficial effect of zinc in chronic cutaneous ulcers is attributed to its anti-inflammatory and antibacterial properties and its ability to enhance reepithelialization. However, there is not enough scientific evidence to make any recommendations for its use in chronic leg ulcers [59, 63].

### 7.6. Behcet's Disease and Oral Aphthosis

Behcet's disease is a vasculopathic condition characterized by recurrent episodes of oral and genital ulcerations with positive pathergy test. Oral aphthae are another troublesome condition of obscure etiology characterized by recurrent painful oral ulcerations particularly in adolescents. Several treatment modalities including corticosteroids and immunosuppressants have been used with variable results. Sharquie et al. [64] in a randomized, controlled, double-blind crossover trial comprising 30 subjects found oral zinc sulphate, 100 mg given thrice daily for three months, to be an effective treatment modality for Behcet's disease without any major adverse effects. They also found oral zinc sulphate (100 mg thrice daily) useful in the treatment of recurrent oral aphthae in another double-blind placebo controlled study of 15 patients [65]. Zinc sulphate had both therapeutic and prophylactic action as it also reduced the relapse rate in recurrent aphthae.

### 7.7. Necrolytic Migratory Erythema (NME)

It is a dermatosis which is usually associated with an underlying pancreatic tumor especially glucagonoma. However, many cases have been described without any underlying pancreatic malignancy. Zinc deficiency is considered a possible reason among many pathogenic hypotheses put forth for this unusual entity as both acrodermatitis enteropathica (inherited zinc deficiency) and acquired zinc deficiency have a striking clinicopathological similarity with necrolytic migratory erythema. It has been also observed that patients of NME have low serum zinc levels and the most consistent improvement is noted with zinc sulfate 440 mg/day [66]. Even in patients with normal serum zinc levels, zinc supplementation leads to clinical improvement of NME [67].

## 8. **Disorders of Hair and Mucosa**

### 8.1. Alopecias

Androgenetic alopecia is a common disorder with an estimated 90% of males above the age of 20 years having some degree of frontal recession. Drugs like minoxidil and finasteride and surgical modalities like hair transplantation form the mainstay of treatment. Zinc has been found to possess antiandrogen action and it modulates 5*α*-reductase type 1 and 2 activity [3]. Although it was less effective as compared to topical 5% minoxidil lotion, a considerable hair growth was observed with topical zinc pyrithione 1% solution in androgenic alopecia in a randomized, investigator-blinded, parallel-group clinical study [68]. Alopecia areata is another common autoimmune disorder with numerous treatment modalities but none is being universally effective. Sharquie et al. [69] in a randomized placebo-controlled, double-blinded crossover study used zinc sulphate in a dose of 5 mg/kg/day in three divided doses for a period of six months and observed a visible clinical response in 62% of patients with alopecia areata. However, there is overall paucity of relevant literature.

### 8.2. Erosive Pustular Dermatosis of Scalp

It is another rare chronic disease manifesting with extensive pustular lesions, erosions, and crusting of the scalp, leading ultimately to scarring alopecia. Response to therapy has been variable with different treatments including topical or systemic antibiotics, oral isotretinoin, or dapsone. Ikeda et al. [70] found oral zinc sulphate to be a safe and effective treatment modality for this uncommon entity.

### 8.3. Seborrhoeic Dermatitis

Seborrhoeic dermatitis is a common entity with an estimated prevalence of 1–3% in the general population. Zinc pyrithione 1% in a shampoo base is a proven treatment modality for seborrhoeic dermatitis and is an active ingredient, mostly in combination with ketoconazole, of several antidandruff shampoos available over the counter or on prescription. It possesses cytotoxic activity against* Pityrosporum ovale* and has antiproliferative action as well which are considered responsible for its clinical efficacy. It also prevents recurrence of flaking, itching, and irritation associated with dandruff and its antifungal activity has been attributed to its ability to disrupt fungal membrane transport by blocking the proton pump that energizes the transport mechanism. However, a combination of zinc pyrithione and ketoconazole is more effective than either agent used alone. Zinc pyrithione 1% in a shampoo base has been found to cause significant reduction in scaling and inflammation but its response was less when compared with 1% ketoconazole [71, 72].

### 8.4. Mucosal (Oral) Lichen Planus

Lichen planus is a chronic inflammatory disease of skin and mucous membranes. Despite plethora of medications, corticosteroids, retinoids, dapsone, and immunosuppressants, a definite cure for lichen planus remains unknown. Mehdipour et al. [73] compared the efficacy of 0.2% zinc mouthwash in combination with fluocinolone with a plain fluocinolone mouthwash in 20 patients of erosive lichen planus. It was observed that pain, irritation, and lesion surface area decreased in both groups. However, decrease in surface area with zinc mouthwash plus fluocinolone was statistically more significant than that with fluocinolone alone.

## 9. **Premalignant and Malignant Dermatoses**

Zinc in high concentration has been found to possess a direct cytotoxic effect and is well known to induce apoptosis of malignant cells and tissue necrosis. This property of zinc has been utilized for its use in premalignant and malignant conditions of skin like xeroderma pigmentosa, actinic keratosis, and basal cell carcinoma. Topical therapy with zinc sulfate solution has been found to have both therapeutic and prophylactic role in patients with xeroderma pigmentosa. Sharquie et al. [74] studied the effect of 20% topical zinc sulphate in 19 patients with xeroderma pigmentosa. Improvement in all types of skin lesions, including softening and lightening of the skin color, and clearance of solar keratosis and small malignancies were observed in 15 patients who continued the study during monthly followup over a follow-up period of 2 years. There was no exacerbation of old lesions or no development of new malignancy. Actinic keratosis, a premalignant condition resulting from proliferation of aberrant epidermal keratinocytes, occurs primarily on sun-exposed skin. Many therapeutic modalities including curettage and cautery, topical agents like 5-fluorouracil, imiquimod (5%) cream, and 3% diclofenac gel, are used for its treatment. Sharquie et al. [75] observed a statistically significant response in the form of clearance of lesions with 25% topical zinc sulphate applied twice daily over the lesions for 12 weeks in 14 of 18 patients. They also observed significant improvement in all 100 lesions of basal cell carcinoma with intralesional 2% zinc gluconate solution without any significant adverse effects in another open-label case interventional study [76]. These beneficial effects of zinc in xeroderma pigmentosa or actinic keratosis are attributable to enhanced wound healing, antioxidant action, sunscreen property, enhanced DNA repair, improved immunity, and accelerated apoptosis of malignant cells [74].

## 10. **Pigmentary Disorders**

Topical zinc has been used for both vitiligo and melasma. Vitiligo is a common depigmenting disorder with variable etiology seen in about 0.1%–2% of the population. As vitiligo patients have been found to have significant low serum zinc levels than normal controls, zinc was postulated to play a role in the management of vitiligo [77, 78]. Yaghoobi et al. [79] in a randomized control trial compared the efficacy of topical steroids alone with the combination of topical steroids and oral zinc sulphate in 15 patients each. An appreciable but statistically insignificant clinical response was observed in 24.7% patients in oral zinc-topical corticosteroid group after four months of therapy as compared to 21.43% in the topical corticosteroid group. Zinc possesses significant antiapoptotic and antioxidant activity and along with other micronutrients like copper and manganese also postulated to play an important role in melanogenesis.

Melasma, a common pigmentary dermatosis, causes significant psychological stress due to cosmetic morbidity in affected patients. It affects all races with a predilection for Hispanics and Asians and accounts for 0.25 to 4% of the patients seen in dermatology practice in Southeast Asia. Genetic predisposition, pregnancy, oral contraceptives, endocrine dysfunction, hormone treatments, or exposure to UV light have been implicated frequently in its pathogenesis. Clinically, it presents in three distinct patterns of centrofacial, malar, and mandibular pigmentation observed in 55–75%, 24–43%, and 1.5–2% patients, respectively, across studies. A large number of treatment modalities have been tried for the treatment of melasma ranging from depigmenting agents like hydroquinone to lasers. Topical zinc sulphate has also been tried in the management of melasma owing to its peeling and sunscreen properties. Sharquie et al. [80] reported a significant reduction in MASI (melasma area and severity index) scores in 14 melasma patients after three months of therapy with 10% topical zinc sulphate without any significant adverse effects. However, this mode of treatment did not find much favor as results could not be reproduced in other studies and no statistically significant improvement was seen with topical zinc therapy [81, 82]. Moreover, it is not cosmetically elegant and acceptability remains poor. Nevertheless, zinc oxide, in micronized forms, remains a common ingredient of most sunscreens used for treatment of melasma.

## 11. **Miscellaneous Dermatoses**

### 11.1. Scars and Keloids

Hypertrophic scars and keloids of any origin are associated with considerable disfigurement. Propensity for recurrences seen with keloids is associated with significant psychological morbidity. Treatment with intralesional corticosteroids, topical silicon gel sheets, surgery, and other physical treatment modalities including lasers and cryotherapy have their own advantages and disadvantages. The beneficial effect of topical zinc in the treatment of keloids in few studies has been attributed to its ability to inhibit lysyl oxidase and stimulate collagenase that leads to decreased production and increased degradation of collagen. Söderberg et al. [83] reported a clinical response in 23 of the 41 patients with keloids after six months of application of a zinc tape. Similarly, Moshref [84] reported a complete clearance of keloids with a very low rate of recurrence in 34% patients with the use of a zinc tape. However, few well-designed studies remain desirable for acceptance of this low cost treatment for this highly distressing condition.

### 11.2. Antiageing

Mahoney et al. [85] evaluated the effects of a bi-metal, 0.1% copper-zinc malonate, containing cream on elastin biosynthesis and elastic tissue accumulation in 21 female patients with photoaged facial skin. After 8 weeks of therapy, significant elastic fiber regeneration was seen in the papillary dermis leading to effacement of wrinkles. The combined photoprotective and elastic regenerative properties of zinc could be used for the development of effective antiageing therapies.

### 11.3. Pruritus

Calamine lotion contains zinc oxide or zinc carbonate and is used frequently for symptomatic relief in pruritus because of its soothing properties. Zinc also inhibits mast cell degranulation and thereby reduces the secretion of histamine, an important mediator of inflammatory response and an inducer of itch, thereby making it a useful treatment option in pruritic conditions [86].

### 11.4. Prevention of Photodamage and Skin Cancers

Zinc oxide is widely used as a broad spectrum physical sunscreen. Its advantage lies in its low cost and an excellent safety profile. It has been used alone and in combination with other physical (titanium oxide) or chemical sunscreen agents. Recently microfine and nano-sized zinc oxide has become available which provides better cosmetic appeal and photoprotection than traditional zinc oxide preparations. Zinc oxide provides protection against UV-A1 (340–380) superior to titanium oxide providing better spectrum protection [87].

## 12. **Comments**

Zinc is an important micronutrient required for the normal function of skin. The daily allowance of elemental zinc in infants with zinc deficiency is usually 3 mg/d for first 6 months and 5 mg/d for second six months. Subsequently, zinc may be supplemented as 10 mg/d during 1–10 years, 15 mg/d for adolescents and adults, and 20–25 mg/d during pregnancy and lactation. For therapeutic purpose zinc is administered orally or parenterally as zinc sulfate (22.5 mg of elemental zinc/100 mg), zinc acetate (30 mg elemental zinc/100 mg), or zinc oxide (80 mg elemental zinc/100 mg). The recommended doses for elemental zinc are 0.5–1 mg/kg/day in divided doses in children and 15–30 mg/day in adults. Gastrointestinal upsets with bloody diarrhea may occur sometimes after ingestion of zinc sulfate beyond recommended doses. Therapeutically, zinc can be used, both topically and in systemic form, for a large number of dermatological disorders. Its efficacy in treating acne perhaps remains the most studied despite varied results. However, it should not substitute the treatment with proven first line therapeutic modalities as most of the studies showing efficacy of zinc are small case series or have small sample size. Interestingly, systemic zinc as a therapeutic modality does not find much favor despite many dermatological conditions shown responding to it. Perhaps more experimental and clinical evidence in the form of appropriately blinded randomized control trials and case-control studies for the treatment of various dermatoses is needed to determine the efficacy of this low cost mode of treatment and compare it with the established treatment modalities. Only after adequate studies for its efficacy and safety, the treatment guidelines or recommendations for zinc therapy can be made. Nevertheless, it can best be used as an adjuvant to established treatment modalities.

## Figures and Tables

**Table 1:** Therapeutic uses of systemic and topical zinc.

Disease	Route of administration	Efficacy	Reference number
Warts	Topical	Efficacious as 5%, 10% zinc sulphate lotion, 20% zinc oxide paste, and 2% intralesional zinc sulphate injection.	[6, 7, 11]
	Oral	10 mg/kg/day oral zinc sulphate for 2 months was an effective modality for recalcitrant warts.	[9, 10]

Cutaneous leishmaniasis	Intralesional	Clinical cure with 2% intralesional zinc sulphate was comparable to meglumine antimoniate.	[12]
	Oral	Oral zinc sulphate in doses of 2.5, 5, and 10 mg/kg/day for 45 days was an effective and safe treatment option.	[13]

Leprosy	Oral	Rapid clinical improvement in leprosy lesions and erythema nodosum leprosum seen on addition of oral zinc along with MDT.	[15, 16]

Herpes genitalis	Topical	Topical 1%, 2%, and 4% zinc sulphate for 3 months was effective in treating and preventing recurrences of herpes genitalis.	[18]

Dermatophytoses	Topical	20% zinc-undecylenate powder applied twice daily for 4 weeks showed clinical improvement in tinea pedis.	[19]

Bromhidrosis	Topical	Topical 15% zinc sulphate solution was efficacious in management of bromhidrosis and foot malodour.	[20, 21]

Pityriasis versicolor	Topical	Topical 15% zinc sulphate solution applied once daily for 3 weeks was effective in pityriasis versicolor.	[22]

Acne vulgaris	Topical	Topical 5% zinc sulphate was effective in mild to moderate acne.	[34, 36]
	Oral	Oral zinc sulphate and zinc gluconate were useful in moderate to severe acne.	[37–40]

Rosacea	Oral	Oral zinc sulphate 100 mg thrice a day was effective in rosacea after 3 months of therapy.	[48]

Hidradenitis suppurativa	Oral	Oral zinc gluconate 90 mg/day showed significant clinical improvement.	[50]

Psoriasis and psoriatic arthritis	Topical	Topical 0.25% zinc pyrithione applied twice daily was found useful in plaque psoriasis.	[52]
	Oral	Oral zinc sulphate was efficacious in psoriatic arthritis.	[53]

Eczemas	Topical	Zinc oxide paste and zinc sulphate were effective in diaper dermatitis and hand eczemas.	[57]

Ulcers	Topical	Topical zinc oxide paste induced rapid healing of vascular and leprosy ulcers.	[60–62]
	Oral	No role of systemic zinc sulphate noted in leg ulcers.	[59]

Behcet's disease and oral aphthae	Oral	Oral zinc sulphate 100 mg/day was an effective in oral aphthosis and Behcet's disease.	[63, 64]

Alopecia areata	Oral	5 mg/kg/day of oral zinc sulphate induced significant hair growth after 6 months of therapy.	[68]

Oral lichen planus	Topical	0.2% zinc mouthwash with fluocinolone was effective in oral lichen planus.	[72]

Xeroderma pigmentosum	Topical	20% zinc sulphate solution applied twice daily cleared the solar keratoses and small malignancies.	[73]

Actinic keratoses	Topical	25% zinc sulphate solution applied twice daily for 12 weeks cleared majority of the lesions.	[74]

Basal cell carcinoma	Intralesional	Intralesional 2% zinc gluconate was efficacious in basal cell carcinoma.	[75]

Vitiligo	Oral	Oral zinc sulphate showed moderate efficacy when given as an adjuvant with topical steroids.	[78]

Melasma	Topical	10% zinc sulphate solution applied twice daily for 3 months showed significant reduction in MASI score.	[79]

Keloids	Topical	Locally applied zinc tape showed significant clearing and reduction in relapses of keloids.	[82, 83]

Antiageing	Topical	0.1% copper-zinc malonate cream applied twice daily for 8 weeks showed significant reduction of wrinkles.	[84]
